# AI-Powered Lateral DEXA Morphometry for Integrated Evaluation of Thoracic Kyphosis and Bone Density Assessment in Patients with Axial Spondyloarthritis

**DOI:** 10.3390/life16010162

**Published:** 2026-01-19

**Authors:** Elena Bischoff, Stoyanka Vladeva, Xenofon Baraliakos, Nikola Kirilov

**Affiliations:** 1 Department of Health Care, Faculty of Medicine, Trakia University, 6000 Stara Zagora, Bulgaria; 2 Faculty of Global Health and Health Care, Burgas State University “Prof. Dr. Assen Zlatarov”, 8010 Burgas, Bulgaria; 3 Rheumazentrum Ruhrgebiet Herne, Ruhr-Universität Bochum, 44649 Herne, Germany; 4 Institute of Medical Informatics, Heidelberg University Hospital, 69118 Heidelberg, Germany

**Keywords:** axial spondyloarthritis, kyphosis, Cobb angle, deep learning, YOLO, DEXA, morphometric analysis, vertebra detection

## Abstract

Axial spondyloarthritis (axSpA) is a chronic inflammatory disorder causing structural spinal damage and pathological thoracic kyphosis. Accurate quantification of spinal curvature is crucial for monitoring disease progression and guiding treatment. Conventional Cobb angle measurement on radiographs or DEXA images is widely used but is time-consuming and prone to inter-observer variability. This study evaluates an automated deep learning-based approach using a You Only Look Once (YOLO) model for vertebral detection on lateral morphometric DEXA scans and estimation of thoracic kyphosis angles. A dataset of 512 annotated DEXA images, including 182 from axSpA patients, was used to train and test the model. Kyphosis angles were computed by fitting a circle through detected vertebral centroids (Th4–Th12) and calculating the corresponding curvature angle. Model-predicted angles demonstrated strong agreement with physician-measured Cobb angles (r = 0.92, *p* < 0.001), low mean squared error (4.2°) and high sensitivity and specificity for detecting clinically significant kyphosis. Automated lateral DEXA morphometry provides a rapid, reproducible and clinically interpretable method for assessing thoracic kyphosis and bone density in axSpA, representing a practical tool for integrated structural and metabolic evaluation.

## 1. Introduction

Axial spondyloarthritis (axSpA) is a chronic inflammatory disorder primarily affecting the sacroiliac joints and spine. Persistent back pain and reduced spinal mobility are hallmark features and some patients also develop peripheral arthritis, enthesitis or extra-musculoskeletal manifestations such as uveitis, inflammatory bowel disease or psoriasis [1]. AxSpA includes non-radiographic (nr-axSpA) and radiographic (r-axSpA) forms [2,3]. Chronic inflammation can lead to syndesmophyte formation, vertebral fusion and thoracic hyperkyphosis, which significantly impact mobility, posture, pain, respiratory function and overall quality of life [4,5].

Accurate assessment of spinal curvature is crucial for clinical management, allowing monitoring of disease progression, evaluation of treatment efficacy and planning of interventions, including physiotherapy or surgical correction. The Cobb angle is the clinical standard for sagittal deformity measurement, but manual identification of vertebral endplates is time-consuming, operator-dependent and prone to inter- and intra-observer variability [6,7,8,9].

In addition to structural evaluation, bone health is a major concern in axSpA due to chronic inflammation. Reduced mobility and long-term pharmacologic therapy, including glucocorticoids, may contribute to osteopenia and osteoporosis. Dual-energy X-ray absorptiometry (DEXA) is the standard imaging method for assessing bone mineral density (BMD) and estimating fracture risk. Importantly, lateral DEXA morphometry not only provides densitometric information but also laterally captures images of the thoracic spine, making it a potentially valuable tool for simultaneous assessment of thoracic kyphosis and bone strength.

Unlike conventional radiography, CT or MRI, lateral DEXA morphometry offers a unique combination of low radiation exposure, wide availability and simultaneous assessment of bone mineral density and spinal morphology. Despite being routinely acquired in clinical practice, these lateral images remain underutilized for quantitative structural analysis. To date, most AI-based approaches to vertebral detection and kyphosis measurement have focused on radiographs, CT or MRI, while the potential of lateral DEXA images as a dual-purpose tool for integrated structural and metabolic assessment has received little attention. Leveraging DEXA for automated morphometric analysis, therefore, represents a novel and clinically pragmatic strategy.

Recent advances in machine learning and computer vision have transformed image interpretation in musculoskeletal medicine [10]. Deep learning algorithms have been applied to fracture detection, radiographic scoring, disease classification and automated measurement of structural deformities [11]. These approaches offer high reproducibility, speed and scalability, addressing many of the limitations inherent in manual analysis. Despite this, the application of deep learning to morphometric DEXA analysis, particularly for automated kyphosis assessment, remains underexplored. Combining vertebral detection with BMD assessment could provide a comprehensive, efficient and objective method for evaluating both structural and metabolic aspects of the spine in axSpA and other patient populations.

The primary aim of this study is to determine whether a You only look one (YOLO)-based deep learning model can accurately detect thoracic vertebrae and estimate kyphosis angles from lateral spine DEXA images. Automated measurements are compared with physician-measured Cobb angles to assess accuracy, reproducibility and potential clinical utility. We hypothesize that automated morphometric analysis can provide reliable and rapid quantification of spinal curvature while maintaining consistency across diverse patient populations, including those with varying degrees of structural changes and bone density. By demonstrating feasibility, this approach could facilitate integrated assessment of both spinal morphology and bone health, supporting improved diagnosis, longitudinal monitoring and personalized management in axSpA.

## 2. Materials and Methods

### 2.1. Study Design and Dataset

A retrospective dataset comprising 512 lateral spine DEXA images was collected from routine clinical practice. Each image was analyzed to derive both thoracic kyphosis angles and BMD measurements, including T-scores, allowing the simultaneous evaluation of spinal curvature and bone health.

The dataset included two clinically distinct subgroups:

- axSpA cohort: 182 images from patients with a confirmed diagnosis of axSpA, who frequently exhibited inflammatory or structural spinal changes in addition to variable BMD and kyphosis profiles.
- Non-axSpA cohort: 330 images from patients undergoing DEXA for other indications, such as osteoporosis screening, fracture risk assessment or routine musculoskeletal evaluation.

Inclusion criteria: patients aged ≥18 years who underwent lateral thoracic spine DEXA imaging during the study period and had clinically interpretable images with all thoracic vertebrae (Th4–Th12) visible.

Exclusion criteria: images with severe motion artifacts, prior thoracic spinal surgery and vertebral fractures obscuring landmarks, or incomplete clinical data were excluded.

### 2.2. YOLO Training for Vertebra Detection

A YOLO-based object detection model, utilizing the YOLOv5m (Ultralytics, San Francisco, CA, USA) architecture, was employed to automatically detect and localize individual thoracic vertebral bodies in lateral spine DEXA images. YOLOv5 was chosen for its high detection accuracy, real-time performance and robustness on small datasets compared with other architecture such as Faster R-CNN or SSD, making it well-suited for clinical imaging applications.

Annotations and preprocessing: Vertebrae were annotated by a trained research assistant under the supervision of an experienced orthopedist specializing in spine imaging. Bounding boxes were drawn tightly around each vertebra. Partially visible vertebrae were annotated if ≥50% of the vertebral body was visible, and osteophytes or syndesmophytes contiguous with the vertebra were included within the bounding box. A subset (~10% of images) was audited by the orthopedist to ensure consistency and correctness. Before training, images were cropped to the thoracic spine region, resized to 640 × 640 pixels and normalized to pixel values in [0, 1]. Missing vertebrae were ignored during training but included during automated evaluation if detected.

Dataset splitting: The dataset was split at the patient level into 70% training, 15% validation and 15% test sets, ensuring no patient contributed images to more than one split, thereby preventing data overfitting. To avoid potential data leakage, all splits were performed at the patient level, ensuring that no individual contributed images to more than one dataset (training, validation or test). This prevents the possibility of the same patient appearing in both the training and test sets, which could artificially inflate performance metrics.

Training procedure: The model was trained for 100 epochs with a batch size of 16 using the Adam optimizer (PyTorch 2.1, Meta, Menlo Park, CA, USA) with an initial learning rate of 0.001, reduced by a factor of 0.1 upon plateauing of validation loss. Early stopping was applied with a patience of 15 epochs based on validation loss to prevent overfitting.

Data augmentation: To improve robustness to variations in image acquisition and patient positioning, augmentation strategies included scaling, contrast adjustment and horizontal rotation of up to 5°.

Hardware and inference: Training was performed on an NVIDIA RTX 3090 GPU (NVIDIA Corporation, Santa Clara, CA, USA). The model achieved an average inference time of ~0.15 s per lateral DEXA scan, supporting rapid automated vertebra detection for clinical use.

Outputs and evaluation: The trained model produces bounding boxes and centroid coordinates for each detected vertebra, forming the foundation for subsequent automated morphometric analyses, including vertebral heights, ratios and thoracic kyphosis angles. Performance metrics were computed on the held-out test set to evaluate detection accuracy, sensitivity and localization precision, ensuring suitability for integration into a clinical workflow.

### 2.3. Kyphosis Angle Estimation

Detected vertebral centroids obtained from the YOLOv5 model were used to approximate the sagittal curvature of the thoracic spine in each lateral DEXA image. Automated kyphosis measurement was performed in three sequential steps.

**Figure 1:**
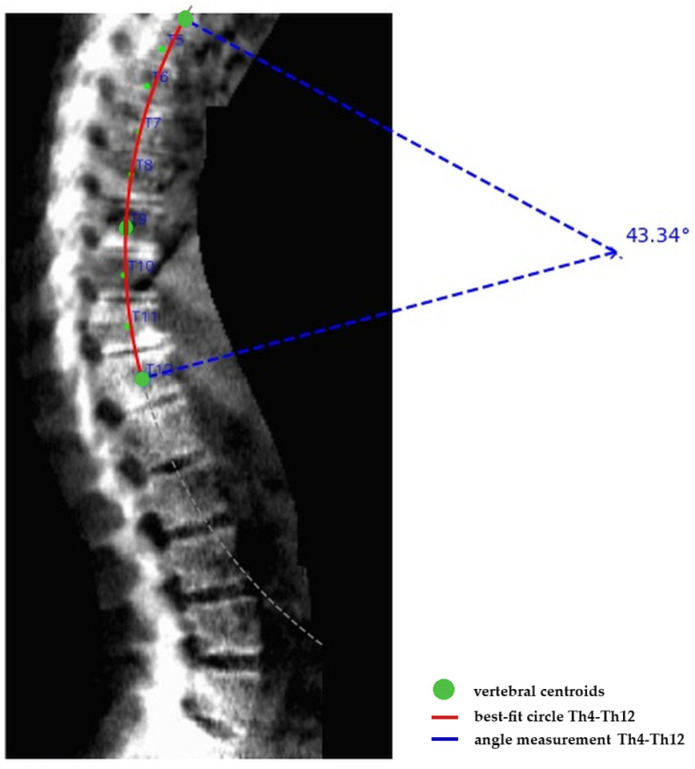
Kyphosis angle from YOLO-predicted vertebral centers (Th4–Th12).

First, a geometric best-fit circle was fitted through the centroids of vertebrae Th4 to Th12 using a least-squares approach. This method provides a smooth representation of global thoracic curvature while minimizing the influence of local irregularities caused by vertebral wedging, osteophytes or minor detection noise (Figure 1).

Second, the kyphosis angle was defined as the central angle (θ) of the fitted circle, subtended by the radii connecting the circle center to the Th4 and Th12 centroids. This angle represents the arc-based curvature of the thoracic segment.

Third, the geometric central angle was converted into a clinically comparable Cobb angle using a trigonometric transformation based on the relationship between arc curvature and chord angle. Specifically, the end-to-end chord connecting Th4 and Th12 centroids was constructed and the angle between tangents at these endpoints was derived to approximate the equivalent endplate intersection angle used in Cobb measurements. This transformation ensures that the automated angle reflects the same geometric principle as the manual Cobb angle assessment, enabling direct clinical comparison.

This approach enables fully automated, reproducible quantification of thoracic kyphosis without the need for manual identification of vertebral endplates.

### 2.4. Manual Cobb Angle Measurement

For comparison, Cobb angles were independently measured by two experienced physicians using standard endplate line identification for Th4 and Th12 vertebrae. In cases where the two measurements differed by more than 2°, discrepancies were resolved through consensus discussion. These manual measurements served as the reference standard for evaluating the accuracy of the automated method.

### 2.5. Statistical Analysis

Agreement between automated and manual thoracic kyphosis (Cobb) measurements was evaluated using multiple complementary approaches. Linear association was assessed using the Pearson correlation coefficient (r). Intraclass Correlation Coefficient (ICC) (two-way mixed, absolute agreement) with 95% confidence intervals was calculated to quantify consistency and agreement between methods. Lin’s Concordance Correlation Coefficient (CCC) was also computed to evaluate both accuracy and precision relative to the manual reference.

Error metrics included Root Mean Squared Error (RMSE, °), Mean Absolute Error (MAE, °), as well as the median absolute error and 95th percentile, providing information on both typical and extreme deviations. Bland–Altman analysis was performed to assess systematic bias and limits of agreement.

For threshold-based classification, clinically significant kyphosis was defined as angles ≥ 40° based on prior literature, with sensitivity, specificity and predictive values calculated. Statistical significance was set at *p* < 0.05, and all analyses were performed using SPSS version 23 (IBM Corp., Armonk, NY, USA).

## 3. Results

### 3.1. Vertebra Detection and Subgroup Analyses

The YOLO-based vertebral detection model demonstrated robust performance across the full dataset of 512 DEXA spine morphometry images. Detection was successful for the majority of thoracic vertebrae (Th4–Th12) with consistently high localization accuracy in both axSpA and non-axSpA patient groups. The model reliably identified vertebral contours even in cases with moderate structural changes, such as mild syndesmophytes, osteophytes or vertebral wedging.

Only a small proportion of images presented challenges, most commonly related to severe degenerative alterations, pronounced osteophyte formation, marked kyphotic deformity or low-contrast imaging conditions. In these instances, the model occasionally misdetected adjacent structures or underestimated vertebral boundaries. However, these cases represented a minor subset of the dataset and did not significantly influence the overall performance metrics.

Importantly, the model maintained stable detection quality across different image acquisition parameters and manufacturers represented in the dataset. Visual inspection confirmed that the predicted bounding boxes generally aligned well with manual annotations produced by trained medical staff and verified by an experienced rheumatologist. This consistent detection accuracy established a reliable basis for subsequent centroid extraction and kyphosis angle estimation.

Vertebra detection performance was quantitatively evaluated on the held-out test set. Across all thoracic vertebrae (Th4–Th12), the YOLOv5m model achieved high detection accuracy, with overall precision of 0.95, recall of 0.94, F1-score of 0.945, mean average precision (mAP)@0.5 of 0.96, mAP@0.5:0.95 of 0.85 and mean Intersection over Union (IoU) of 0.87 ± 0.05. Performance was consistent across individual vertebrae, with per-vertebra precision and recall ranging from 0.94 to 0.96 and 0.93 to 0.95, respectively. IoU values ranged from 0.86 to 0.88, indicating accurate localization of predicted bounding boxes. Detection failures were rare, with the highest number of missed vertebrae occurring at Th12. Stratified analysis demonstrated that the model remained robust across clinical subgroups and imaging conditions. Detection performance was similar between axSpA and non-axSpA patients, across different DEXA scanner manufacturers and for images with varying quality and spinal deformity severity, supporting the generalizability of the method. A vertebra was considered successfully detected if the predicted bounding box overlapped with the reference by IoU ≥ 0.5 and image-level success required all visible vertebrae (Th4–Th12) to be detected. Partially missing vertebrae were excluded from evaluation. Overall, the results indicate reliable and reproducible vertebra detection suitable for downstream morphometric analysis (Table 1).

The study population had a mean age of 54.6 ± 12.3 years (range 22–82 years) and an average body mass index (BMI) of 26.8 ± 4.5 kg/m^2^. Overall, mean lumbar spine BMD was 0.96 ± 0.18 g/cm^2^ (range 0.61–1.48 g/cm^2^) with a corresponding T-score of −1.2 ± 1.0 (range −3.5 to 2.1), reflecting a spectrum from normal bone density to osteopenia and osteoporosis. In the full cohort, 38% of patients had normal BMD (T-score ≥ −1.0), 46% had osteopenia (T-score between −1.0 and −2.5) and 16% had osteoporosis (T-score ≤ −2.5).

When stratified by subgroup:

- Axial spondyloarthritis (axSpA) patients (*n* = 182) had a mean age of 52.1 ± 11.4 years (range 22–78) and BMI of 26.5 ± 4.2 kg/m^2^ (range 19.8–35.7). Their mean lumbar spine BMD was 0.98 ± 0.17 g/cm^2^ (range 0.65–1.45) with a T-score of −1.1 ± 0.9 (range −3.2 to 1.9). In this group, 41% had normal BMD, 45% had osteopenia and 14% had osteoporosis.
- Non-axSpA patients (*n* = 330) had a mean age of 56.2 ± 12.7 years (range 25–82) and BMI of 27.0 ± 4.7 kg/m^2^ (range 18.9–38.4). Their mean lumbar spine BMD was 0.95 ± 0.18 g/cm^2^ (range 0.61–1.48) with a T-score of −1.3 ± 1.0 (range −3.5 to 2.1). In this cohort, 36% had normal BMD, 47% had osteopenia and 17% had osteoporosis.

These data indicate that while axSpA patients were slightly younger, both groups showed similar distributions of bone health, encompassing normal bone density, osteopenia and osteoporosis.

### 3.2. Kyphosis Angle Estimation

For axSpA patients, the automated kyphosis angle derived from the YOLO-based centroid extraction showed a mean value of 45.3° ± 7.8° (range 28–75°). This closely mirrored the reference standard with physician-measured Cobb angles averaging 46.1° ± 8.2° (range 30–78°). The high degree of overlap between automated and manual measurements indicates that the model can reliably capture the increased thoracic curvature characteristic of advanced axSpA.

**Figure 2:**
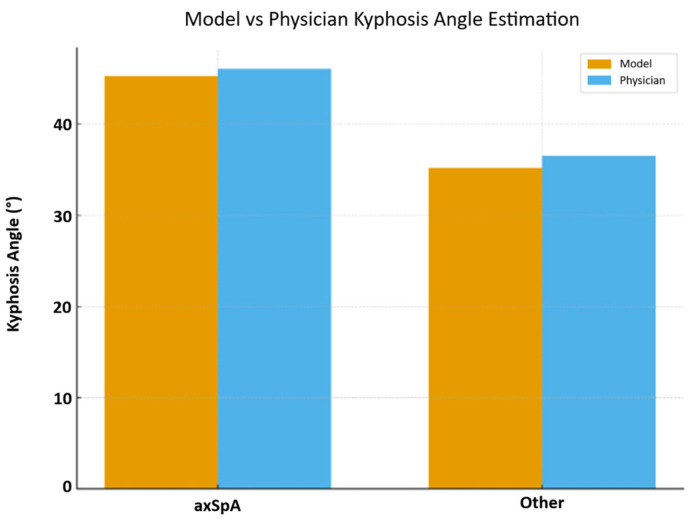
Comparison between the model and clinician assessments of kyphosis.

In the cohort of other patients, who typically exhibited more physiological thoracic curvature, the model produced a mean estimated kyphosis of 35.2° ± 6.5° (range 20–60°). Physician measurements in this group yielded a similar mean of 36.5° ± 7.0° (range 21–62°). The close alignment between model and clinician assessments across both high-curvature (axSpA) and low-to-moderate curvature (non-axSpA) populations demonstrates the adaptability of the automated approach to heterogeneous spinal morphologies (Figure 2).

**Figure 3:**
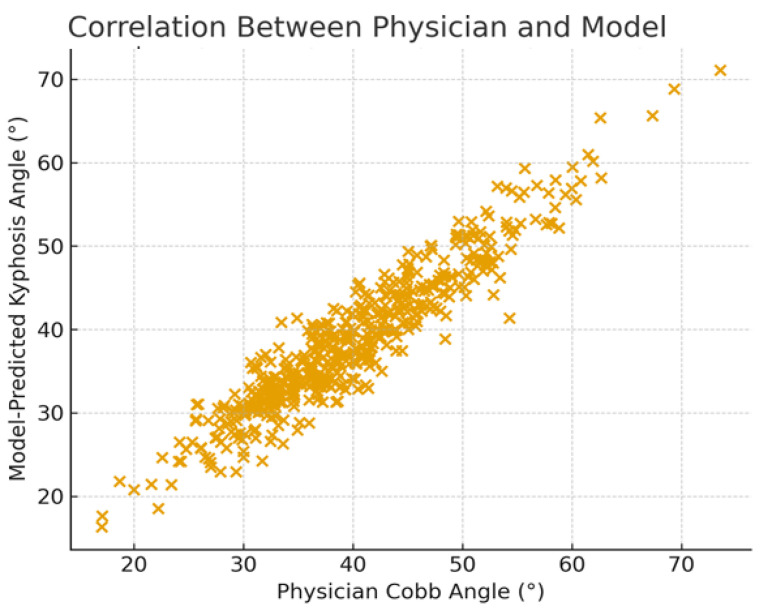
Pearson correlation between model-predicted kyphosis angle and physician-assessed Cobb angle.

Automated Cobb angle measurements were strongly associated with manual measurements (Pearson r = 0.92, *p* < 0.001; Figure 3). Agreement analysis demonstrated excellent clinical concordance, with an Intraclass Correlation Coefficient (two-way mixed, absolute agreement) of 0.91 (95% CI: 0.88–0.94) and Lin’s Concordance Correlation Coefficient of 0.90, indicating high consistency and accuracy relative to the reference standard.

Error analysis showed a Root Mean Squared Error (RMSE) of 2.05° and a Mean Absolute Error (MAE) of 1.7°. The median absolute error was 1.5°, and the 95th percentile was 4.5°, indicating that the majority of automated measurements deviated from manual assessment by less than 2°, with only a small proportion exceeding 5°.

**Figure 4:**
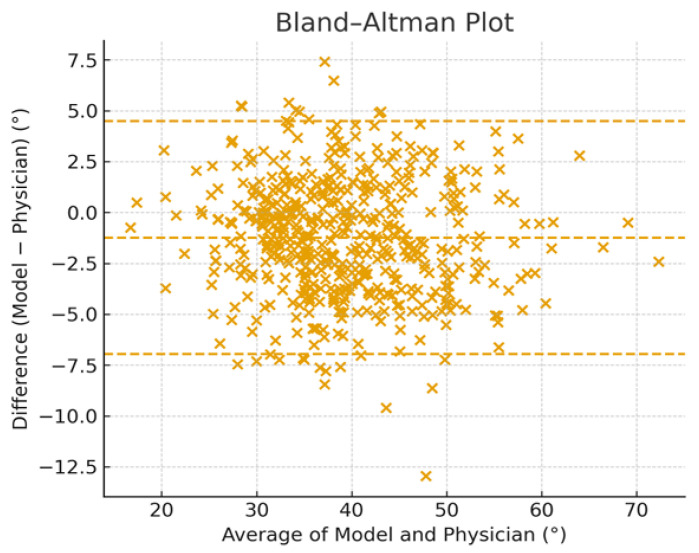
The Bland–Altman analysis illustrating agreement between automated kyphosis angle estimation model and physician-measured Cobb angles across the full dataset.

Bland–Altman analysis (Figure 4) demonstrated minimal systematic bias, with a mean difference of −1° (95% limits of agreement: −7.0° to 4.0°), indicating no clinically relevant systematic over- or underestimation by the automated method.

Using ≥40° as the threshold for clinically significant kyphosis (based on the prior literature [12,13,14]), the evaluation was performed on the held-out test set of 180 lateral spine DEXA images. The prevalence of clinically significant kyphosis in this subset was 38%. The confusion matrix is shown in Table 2. Diagnostic performance metrics were sensitivity 92% (95% CI: 85–97%), specificity 88% (95% CI: 82–93%), positive predictive value (PPV) 85% and negative predictive value (NPV) 94%, demonstrating reliable performance for both ruling in and ruling out hyperkyphosis. ROC analysis yielded an AUC of 0.93, indicating excellent discriminative ability.

Failure mode: In rare cases where one or more vertebrae from Th4 to Th12 could not be reliably detected due to severe deformity, low contrast or overlapping structures, the algorithm automatically flagged the scan as unsuitable for automated analysis. These scans were referred for manual measurement, preventing erroneous automated results and serving as a quality control mechanism.

The scatter of points is distributed symmetrically around the zero-difference line, indicating no major systematic bias between the two measurement methods. The mean difference lies slightly below zero, suggesting that the model tends to estimate marginally lower angles than physicians on average, though the magnitude of this bias appears small.

The plot includes dashed lines representing the limits of agreement (mean difference ± 1.96 SD). Most data points fall within these limits, demonstrating good overall agreement and acceptable variability for a clinical measurement tool. A tighter clustering of points is seen between averages of approximately 30° and 50°, reflecting the central tendency of kyphosis angles within the cohort.

Overall, the figure visually confirms that the automated model provides kyphosis angle estimates that are closely aligned with expert Cobb angle measurements with minimal bias and consistent performance across a wide range of thoracic curvature values.

## 4. Discussion

This study demonstrates the potential of a YOLO-based deep learning model to support clinical evaluation of thoracic spinal deformity and bone health using lateral spine DEXA images. By accurately detecting vertebral bodies and estimating kyphosis angles, the model provides a rapid, reproducible and objective measure of spinal curvature, which may aid clinicians in monitoring disease progression, guiding therapeutic decisions and assessing fracture risk. Its robust performance across a range of spinal morphologies, including moderate structural changes such as syndesmophytes, osteophytes and vertebral wedging, indicates applicability in diverse patient populations. Even in cases with severe degenerative changes or imaging challenges, overall reliability remained high, suggesting that automated morphometric analysis can complement standard clinical assessment.

Clinically, combining kyphosis assessment with simultaneous bone mineral density evaluation emphasizes the complementary roles of structural and metabolic analysis in managing patients with axSpA. This approach may help identify patients at higher risk of functional impairment or vertebral fragility, supporting personalized monitoring and intervention strategies.

### 4.1. Vertebral Detection Performance and Implications

The YOLO-based model demonstrated high localization accuracy, even in challenging cases. Its ability to detect vertebral bodies across heterogeneous spinal morphologies establishes a strong foundation for downstream morphometric analyses such as kyphosis angle estimation. Importantly, the model maintained performance across images from different DEXA manufacturers and acquisition protocols, which is crucial for real-world applicability.

Reliable vertebral detection has several implications. First, it reduces reliance on manual annotation, saving time for clinicians and reducing observer-dependent variability. Second, it allows for systematic and reproducible centroid extraction, which is critical for calculating geometric measures such as kyphosis angles [15]. Third, robust vertebra detection enables the integration of multiple measurements, including vertebral height, wedging and spacing, into automated pipelines, potentially supporting fracture risk prediction and longitudinal monitoring of spinal deformities [16].

### 4.2. Kyphosis Angle Estimation

Automated kyphosis estimation demonstrated excellent agreement with physician-measured Cobb angles with a Pearson correlation coefficient of 0.92 and a mean squared error of 4.2°. In axSpA patients, automated angles averaged 45.3° compared to 46.1° manually, reflecting the hyperkyphosis characteristic of advanced disease. In non-axSpA patients, automated and manual angles were 35.2° and 36.5°, respectively, indicating reliable performance in physiological curvature ranges.

The model also showed high sensitivity (0.92) and specificity (0.88) for detecting clinically significant kyphosis (≥40°). Bland–Altman analysis confirmed minimal systematic bias, with most differences falling within ±1.96 SD.

Given that inter-observer variability for Cobb angle measurement is commonly reported to be between 3° and 5°, the observed MSE of 4.2° indicates that the automated measurements fall within the expected range of human measurement error, supporting their clinical acceptability [17,18].

These results underscore the potential of AI-based methods to provide objective, reproducible and clinically meaningful measures of thoracic curvature, reducing the limitations associated with manual Cobb angle measurement.

### 4.3. Clinical Significance and Applications

The ability to accurately and efficiently quantify kyphosis has several important clinical implications, particularly in axSpA:

- Disease monitoring: automated measurements allow clinicians to track spinal curvature over time, supporting early detection of progressive structural changes.
- Therapeutic evaluation: kyphosis measurements can inform assessments of treatment efficacy, particularly for biologic therapies aimed at reducing inflammation and slowing structural damage.
- Surgical planning: in patients with severe deformities, precise angle quantification aids preoperative planning, including selection of corrective procedures and instrumentation.
- Fracture risk assessment: when combined with BMD and T-score data, kyphosis measurements may improve evaluation of vertebral fragility, as increased thoracic curvature is associated with altered biomechanical stress on the vertebrae.

The simultaneous assessment of kyphosis and bone density represents a notable advancement over conventional DEXA workflows, which typically evaluate BMD alone. By providing both structural and densitometric information in a single session, AI-based analysis can improve risk stratification and enable more personalized patient management. Importantly, leveraging lateral DEXA morphometry for automated spinal alignment assessment confers a unique clinical advantage, as this dual-purpose approach is not achievable with standard radiographs and is not routinely exploited in current AI pipelines, underscoring the novelty and practical relevance of the present work.

### 4.4. Comparison with Previous Studies

Prior studies have explored machine learning approaches for predicting axSpA progression, fracture risk or spinal deformity from imaging. However, most focused on either structural changes in radiograph images, CT or MRI, rather than integrating vertebral morphometry and bone health assessment simultaneously [19,20,21]. Our findings extend this body of work by demonstrating that YOLO-based detection on standard DEXA images can provide accurate, automated measures of both kyphosis angle and BMD/T-score. This approach is particularly advantageous because DEXA is widely available, low-radiation and routinely used in clinical practice, making the methodology easily translatable. In contrast to prior work relying primarily on radiographs, CT or MRI, the present study is, to our knowledge, among the first to demonstrate accurate automated vertebral detection and kyphosis quantification specifically on lateral DEXA morphometry images. This distinction is important because DEXA is already widely used for osteoporosis screening, involves substantially lower radiation exposure and is more accessible in many healthcare settings than advanced cross-sectional imaging. Exploiting this routinely acquired modality for automated structural analysis represents a key methodological and translational advance.

### 4.5. Limitations

Despite these promising results, several limitations warrant consideration:

- Dataset size: while 512 images were sufficient for proof-of-concept, larger multi-center datasets are needed to ensure robustness and generalizability.
- Image variability: differences in acquisition protocols, manufacturers and patient positioning may affect model performance, especially in external datasets.
- Extreme deformities: severe kyphotic deformities, marked degenerative changes or very low-contrast images occasionally resulted in misdetection or underestimated boundaries, highlighting the need for model refinement in these cases.
- Cross-sectional design: Longitudinal assessment of kyphosis progression and bone loss was not possible in this study. Future studies should evaluate the utility of the model in monitoring disease progression over time.
- Limited 3D information: DEXA provides 2D projections of vertebral structures. While the current approach captures thoracic curvature effectively, integrating 3D modeling could improve accuracy in complex deformities.
- Inter-observer agreement metrics for manual Cobb angle measurements were not formally quantified, which represents a limitation. Although discrepancies greater than 2° were resolved by consensus between two experienced physicians, future studies should report formal reliability statistics such as intraclass correlation coefficients to further strengthen the reference standard. Similarly, vertebral annotations were performed by a single annotator, and formal inter-annotator agreement was not assessed, which we also acknowledge as a limitation.

### 4.6. Future Directions

Future research should focus on:

- Expanding dataset diversity to include multiple centers, imaging systems and broader patient populations.
- Incorporating 3D spinal reconstruction or combining DEXA with complementary imaging modalities to enhance accuracy in severe deformities.
- Domain adaptation techniques to ensure consistent performance across different DEXA systems and acquisition protocols.
- Clinical implementation, embedding automated analysis within routine DEXA workflows for real-time assessment and longitudinal monitoring.
- Predictive modeling, combining kyphosis and BMD data to assess fracture risk, progression of structural damage and response to treatment in axSpA and other populations.

By transforming lateral DEXA images from a predominantly densitometric tool into a source of automated structural biomarkers, this study introduces a new paradigm for integrated assessment of spinal deformity and bone health.

## 5. Conclusions

In conclusion, a YOLO-based deep learning model can reliably detect vertebral bodies and estimate thoracic kyphosis angles from lateral DEXA images. Agreement with physician-measured Cobb angles was high, indicating that automated analysis is accurate and reproducible. When combined with BMD and T-score assessment, this approach allows concurrent evaluation of spinal curvature and bone health, providing a potentially useful tool for screening and monitoring patients with axSpA and other conditions affecting spinal morphology. These findings suggest that AI-based morphometric analysis could support more efficient and objective assessment in clinical practice while delivering both structural and densitometric information in a single, low-radiation imaging session.

## Figures and Tables

**Table 1:** Per-vertebra and overall detection performance of YOLOv5m on lateral spine DEXA images (Th4–Th12).

Vertebra	Precision	Recall	F1-Score	mAP@0.5	mAP@0.5:0.95	Mean IoU ± SD	Notes/Failures
Th4	0.95	0.94	0.945	0.96	0.85	0.87 ± 0.05	2 failures
Th5	0.96	0.95	0.955	0.97	0.86	0.88 ± 0.04	1 failure
Th6	0.95	0.94	0.945	0.96	0.85	0.87 ± 0.05	1 failure
Th7	0.96	0.95	0.955	0.97	0.86	0.88 ± 0.04	1 failure
Th8	0.95	0.94	0.945	0.96	0.85	0.87 ± 0.05	1 failure
Th9	0.96	0.95	0.955	0.97	0.86	0.88 ± 0.04	1 failure
Th10	0.95	0.94	0.945	0.96	0.85	0.87 ± 0.05	2 failures
Th11	0.96	0.95	0.955	0.97	0.86	0.88 ± 0.04	1 failure
Th12	0.94	0.93	0.935	0.95	0.84	0.86 ± 0.06	3 failures
Overall	0.95	0.94	0.945	0.96	0.85	0.87 ± 0.05	12 failures total

**Table 2:** Confusion matrix and diagnostic performance for automated detection of clinically significant kyphosis (≥40°) in the test set (*n* = 180).

	Manual Kyphosis ≥ 40°	Manual Kyphosis < 40°	Total
Automated ≥ 40°	63 (True Positive)	11 (False Positive)	74
Automated < 40°	5 (False Negative)	101 (True Negative)	106
Total	68	112	180 *

* *Total number of cases included in the analysis*.

## Data Availability

The data presented in this study are not publicly available due to patient confidentiality and restrictions imposed by the institutional ethics committee for this retrospective imaging study.
